# “*Private Hospitals Generally Offer Better Treatment and Facilities*”: Out-of-Pocket Expenditure on Healthcare and the Preference for Private Healthcare Providers in South India

**DOI:** 10.3390/ijerph21101287

**Published:** 2024-09-26

**Authors:** Sagarika Kamath, Mahalakshmi Poojary, Harshith Shetty, Kshithija Umesh, Soham Kar, Vani Lakshmi Ramesh, Gaurav Hajare, Albi Thomas, Helmut Brand, Selim Jahangir, Rajesh Kamath

**Affiliations:** 1 Department of International Health, Care and Public Health Research Institute—CAPHRI, Faculty of Health, Medicine and Life Sciences, Maastricht University, 6200 MD Maastricht, The Netherlands; dr.sagarikarkamath@gmail.com (S.K.); helmut.brand@maastrichtuniversity.nl (H.B.); 2 Department of Social and Health Innovation, Prasanna School of Public Health, Manipal Academy of Higher Education, Manipal 576104, Karnataka, India; mahalakshmi.psphmpl2022@learner.manipal.edu (M.P.); harshith.psphmpl2022@learner.manipal.edu (H.S.); kshithija.psphmpl2022@learner.manipal.edu (K.U.); soham.psphmpl2022@learner.manipal.edu (S.K.); 3 Department of Data Science, Manipal Academy of Higher Education, Manipal 576104, Karnataka, India; vani.lakshmi@manipal.edu; 4 Directorate of Online Education, Manipal Academy of Higher Education, Manipal 576104, Karnataka, India; hajare.gaurav@manipal.edu (G.H.); albi.thomas@manipal.edu (A.T.); 5 Department of Health Information, Prasanna School of Public Health, Manipal Academy of Higher Education, Manipal 576104, Karnataka, India

**Keywords:** out-of-pocket expenditure, healthcare access, affordability, government hospital, private hospitals, insurance, India

## Abstract

Out-of-pocket expenditure (OOPE) directly reflects households’ financial burden for healthcare. Despite efforts to enhance accessibility and affordability through government initiatives and insurance schemes, OOPE remains problematic, especially in rural areas with inadequate public healthcare infrastructure. This study examines factors influencing OOPE in Karnataka’s Dakshina Kannada, Udupi, and Shimoga districts, investigating socioeconomic characteristics, healthcare infrastructure, and accessibility to inform policies for equitable healthcare access and reduced household financial strain. Using purposive sampling, 61 semi-structured interviews were conducted in rural and urban South Karnataka, recorded in Kannada after obtaining consent, and thematically analyzed. Results revealed mixed perceptions of healthcare quality, cost, and accessibility between government and private hospitals. Government facilities were lauded for improved infrastructure and affordability, while private hospitals were preferred for quality and personalized care despite higher costs. Health insurance significantly impacted OOPE reduction. Participants emphasized the need for increased awareness of government insurance programs and improved quality in public hospitals. The study concludes that private hospitals are favored for superior care despite expenses, while government hospitals are valued for affordability. Expanding insurance coverage and improving public awareness are crucial for enhancing healthcare accessibility and affordability.

## 1. Introduction

**Figure 1:**
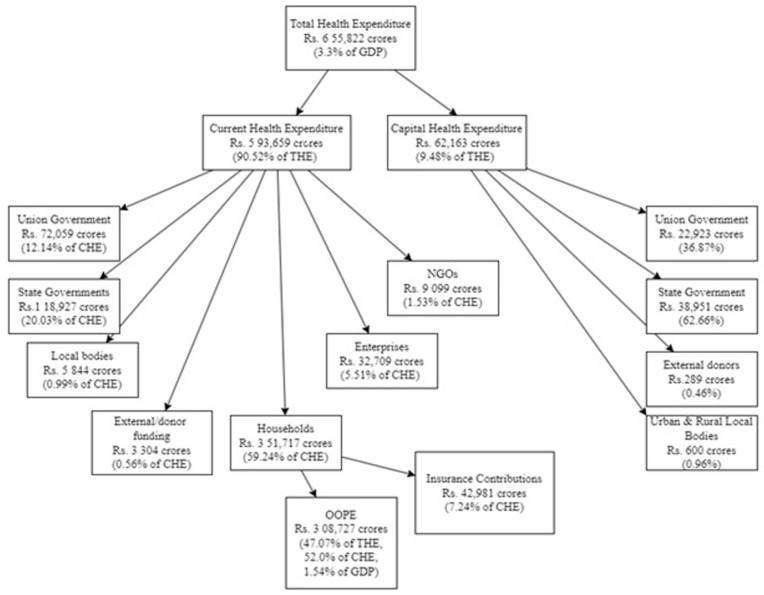
THE Breakdown for 2019–2020.

In India, a substantial proportion (47.1%) of the Total Healthcare Expenditure (THE) fell under Out-of-Pocket Expenditures (OOPEs) in 2019–2020, which is the direct financial burden borne by households for healthcare services [1]. This phenomenon emerges from a confluence of factors characterizing India’s healthcare system: a co-existence of public and private healthcare facilities, inadequate public healthcare infrastructure, high medical costs, limited population-wide health insurance coverage, geographically skewed access to healthcare services, and a growing reliance on private health facilities. The disparity is particularly pronounced between rural and urban settings. While 72% of the population resides in rural regions, approximately 80% of physicians, 75% of dispensaries, and 60% of hospitals are concentrated in urban areas [2]. OOPE plays a critical role in healthcare financing, particularly in low- and middle-income countries, where it often constitutes more than half of THE [3]. These expenditures represent the direct costs incurred by individuals for various healthcare services, encompassing preventive, curative, rehabilitative, palliative, or long-term care needs. Individuals typically utilize household income including remittances, savings, or even loans to cover these expenses. It is crucial to distinguish OOPE from healthcare costs reimbursed by third-party payers, such as government health programs and public or private health insurance providers. This is essential for analyzing the true financial burden borne by individuals [4]. Despite a significant decline, OOPE in India remains a substantial burden compared to the global average (18.1% of THE as of 2019) [5]. The financial strain of OOPE has demonstrably increased at the household level from 64% (2005) to 81% (2012) in rural areas and 65% (2005) to 78% (2012) in urban areas. Furthermore, the share of OOPE relative to total household expenditure is consistently higher in rural areas (6.34% in 2005 and 7.73% in 2012) compared to urban areas (5.05% in 2005 and 5.74% in 2012) [6]. The composition of OOPE highlights specific cost drivers like pharmaceutical expenses, which account for 40% to 50% of OOPE. Diagnostic tests contribute approximately 10%, while medical consultation fees account for roughly 13%. Additionally, non-medical expenditures, such as transportation and accommodation costs, have witnessed a substantial increase, rising from 7% to 17% of OOPE over the past two decades [7]. The financial burden of OOPE can become catastrophic when it exceeds 10% of a household’s total consumption expenditure [8]. High OOPE pushes an estimated 3% of the Indian population (approximately 38 million individuals) below the poverty line annually [9]. The achievement of Universal Health Coverage (UHC) hinges on robust health systems that prioritize a trifecta of objectives: financial sustainability, high-quality care delivery, and equitable access for all citizens. Policy interventions must, therefore, focus on cost-containment strategies for essential healthcare services to ensure equitable utilization. The Government Health Expenditure (GHE) as a proportion of GDP has increased to 1.3% in FY19 from 1.2% in FY14. Furthermore, the GHE’s share in THE has grown significantly from 28.6% in FY14 to 40.6% in FY19. India’s THE for FY19 was Rs. 5,96,440 crore (Rs. 4470 per capita, 3.2% of GDP). Of this, capital expenditure represents Rs. 56,194 crore (9.4% of THE), with Current Health Expenditure (CHE) constituting the remaining Rs. 5,40,246 crore (90.6%). In total, 34.3% of GHE is contributed by the Union Government and 65.7% by State Governments [10]. Since 2007, the Indian government has undertaken a commendable effort to expand UHC through the implementation of Publicly Funded Health Insurance Schemes (PFHISs) at both central and state levels, specifically targeting marginalized and underprivileged populations. However, a significant portion of Indian households continue to rely on OOPE despite the existence of social security programs like the Pradhan Mantri Bhartiya Janaushadhi Pariyojana (PMBJP) which aims to provide affordable, high-quality medicines, alongside health insurance schemes like Ayushman Bharat Pradhan Mantri Jan Arogya Yojana (AB-PMJAY), Central Government Health Scheme (CGHS), Employees’ State Insurance Scheme (ESIS), Mahatma Jyoti Rao Phule Jan Arogya Yojana (MJPJAY), and Vajpayee Arogyashree Scheme (VAS) [11]. Inadequate public health infrastructure, coupled with high medical expenses and limited insurance coverage, incentivizes patients to seek care from the more expensive private sector, often perceived as offering superior service quality, further exacerbating the burden of OOPE [12]. India allocated 3.3% of its GDP (Rs. 4863 per capita at constant prices) to healthcare in FY19. CHE constituted approximately 90.5% of THE. A more detailed breakdown of THE is depicted in Figure 1 [13,14,15].

**Figure 2:**
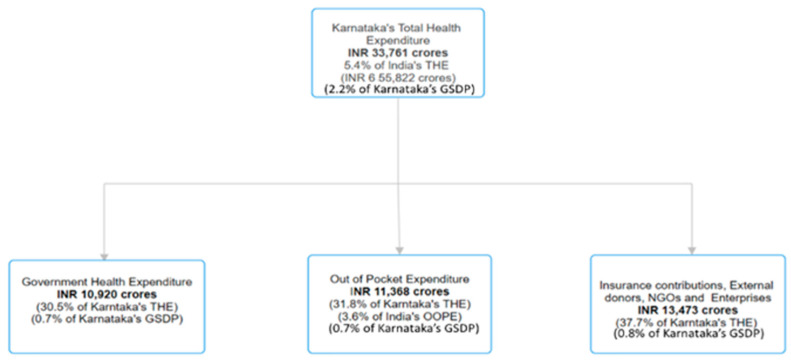
THE Breakdown of Karnataka for 2019–2020.

An examination of Karnataka’s health financing in the same year reveals that the GHE remains relatively modest at Rs. 10,920 crores, representing less than 1% of the Gross State Domestic Product (GSDP) of Rs. 16.1 lakh crore while the state’s THE was Rs. 33,761 crores (constituting 5.4% of India’s THE). Furthermore, OOPE by individuals for healthcare services amounted to Rs. 11,368 crores, exceeding GHE and accounting for 31.8% of Karnataka’s THE. This highlights a significant dependence on private financing for healthcare within the state. Beyond government spending and OOPE, contributions from health insurance, external donors, NGOs, and enterprises collectively reached Rs. 13,473 crores, representing 37.7% of Karnataka’s THE. A detailed breakdown of these financing mechanisms is presented in Figure 2 [13,14,15].

This suggests a need for increased attention to resource allocation to strengthen the healthcare infrastructure and accessibility in the state [13,14,15]. A significant number of Indian households continue to experience OOPE in healthcare despite the enrollment of 70% of the population in some form of health insurance [16]. This disparity is further amplified in Karnataka, where only 28% of households have health insurance coverage for at least one member [17]. A growing reliance on costlier private healthcare services compounds this financial challenge, contributing to elevated rates of OOPE. Private hospitals manage a substantially higher proportion of hospitalization cases in the state, handling 66% of cases in rural areas and 81% in urban areas. This trend mirrors national patterns, where private hospitals attend to 52% of hospitalization cases in rural India and 61% in urban India [18]. As of 2019–2020, rural Karnataka’s healthcare infrastructure comprises 8891 Sub-Centers (SCs), 2141 Primary Health Centers (PHCs), and 182 Community Health Centers (CHCs). However, this network faces a significant shortfall of 329 CHCs according to PHFI norms, as seen in Table 1 [19]. The limited availability of public facilities like CHCs equipped for secondary care or advanced treatments could deter patients from utilizing this level of care within the public system. Consequently, they may be compelled to seek services from private providers, potentially incurring higher OOPE.

Table 2 reveals a significant disparity in OOPE for inpatient treatment between rural and urban Karnataka, across public and private health facilities. In both rural and urban regions, OOPE for inpatient care is considerably lower in public institutions compared to private facilities. Medicines constitute a substantial portion of inpatient OOPE, accounting for 49% and 51% of expenditure in rural and urban public health facilities, respectively. Diagnostics follow closely, representing 18% and 20% of inpatient medical expenditure in rural and urban areas, respectively [19].

In addition, the United Nations’ sustainable development goals on health (SDG-3) also focus on the accessibility of affordable high-quality healthcare for all without financial burden [20]. India is facing a triple burden of diseases: a rise in the incidence of injuries, an unresolved backlog of infectious diseases, and an increase in non-communicable diseases (NCDs) [21]. Moreover, the high OOPE influences healthcare access, food consumption and nutrition, and puts households at risk of financial burden and impoverishment [22]. However, limited literature focuses on the reasons for OOPE in India. Previous studies have focused on hospitalization, mental health issues, NCDs, cancer, diabetes, and tuberculosis for OOPE in India [23,24,25,26,27,28]. Against this backdrop, the present qualitative study investigates the factors of preferring private hospitals over government hospitals that contribute to OOPE on healthcare across rural and urban households in three districts of Karnataka, India. The Dakshina Kannada district population exceeding 2.3 million has a comparable urban–rural divide (47% rural). Public healthcare utilization remains low, particularly in rural areas. This can be attributed to factors such as limited-service availability, provider proximity, socioeconomic status of residents, and perceptions of service quality. Interestingly, high-income households may utilize public facilities when confident in the quality of care, highlighting the multifaceted nature of healthcare access and affordability [29]. The Udupi district has a population of 1.2 million people (62% rural), a low poverty rate at 9.9%, and an 86% college education rate. The average annual OOPE per household is Rs. 5000, compared to an annual income of Rs. 1,000,000. Despite positive economic indicators, OOPE remains high due to a variety of issues including poverty, health insurance penetration, high cost of private healthcare options, and limited access to competent public healthcare facilities [30,31,32,33,34,35]. The Shimoga district, with a population of 1.87 million (64.41% rural), demonstrates a similar pattern. Low health insurance prevalence (26%) and limited access to quality medical care in rural areas push households towards expensive private providers, further exacerbating their OOPE [35]. Within this framework, the current research investigated reasons for choosing private healthcare versus public healthcare, as well as out-of-pocket costs. These expenses, which are classified as direct charges for services like consultation fees, prescription drugs, diagnostic tests, and hospital stays, significantly impact low-income households. Perceived quality, shorter wait times, specialized services, and the professional demeanor of healthcare staff are the main factors influencing consumer preference for private healthcare. These variables affect the availability and use of healthcare, which raises expenses and exacerbates financial vulnerability and health inequities.

Healthcare out-of-pocket expenses (OOPEs) are a major global concern, especially in low- and middle-income countries (LMICs) with inadequate healthcare finance systems. OOPE is cited by the World Health Organization (WHO) as a major cause of poverty and financial hardship. Over 930 million people worldwide have experienced financial difficulties when receiving health care in 2019, and approximately 150 million people are affected by catastrophic health costs each year [36]. The burden of OOPE is especially noticeable in countries with inadequate public healthcare systems, where there is a heavy dependence on private healthcare providers, increasing household direct payments. Households face two additional challenges that exacerbate their financial burden: the cost of healthcare associated with illness and lost wages resulting from missed work [37].

Social health insurance and tax-based funding greatly lower OOPE in high-income countries, ensuring that access to healthcare is not restricted by financial constraints [38]. However, OOPE accounts for a sizable share of overall health spending in LMICs, where insurance coverage is often limited, and health systems are inadequate. According to studies conducted in nations like Bangladesh, Vietnam, and Nigeria, households usually turn to debt or asset sales as a means of paying for medical expenses, which creates unstable financial situations [39,40,41].

Sub-Saharan African research indicates that the lack of accessible and inadequate public health facilities frequently makes OOPE worse by pushing people to seek care in the more costly private sector [42]. Similarly, OOPE in Latin America is still high because of coverage gaps and the expense of uninsured care, even if insurance coverage has increased in countries such as Mexico and Brazil [43]. 

Out-of-pocket expenditure (OOPE) for healthcare has been widely studied in India and other South Asian countries, with the majority of research employing quantitative methods, such as household surveys and econometric analyses, to assess the financial burden on individuals and households [44]. OOPE significantly contributes to impoverishment and catastrophic health expenditure in the region [45,46]. However, qualitative methods are rarely used to explore the contextual and experiential dimensions of OOPE. Studies focusing on qualitative approaches, such as interviews or focus groups, to understand the lived experiences of households dealing with healthcare costs are notably scarce.

### Innovations and Contributions of the Study

This research adds to the body of knowledge by offering an understanding of the underlying factors impacting OOPE in the particular setting of Karnataka, a state with a wide range of healthcare issues. This study dives into the contextual elements, such as the function of healthcare infrastructure and socioeconomic features in shaping OOPE, in contrast to prior studies that frequently offer a broad perspective. Three districts—Dakshina Kannada, Udupi, and Shimoga—are the focus of the study, which provides region-specific insights essential for developing focused policy interventions to address the OOPE. The study also highlights the importance of health insurance in reducing out-of-pocket expenses (OOPE), an aspect that is frequently disregarded in the literature on healthcare expenses in India.

## 2. Materials and Methods

The study is based on qualitative methods, particularly in-depth interviews. The data were obtained by interviewing participants to assess reasons for out-of-pocket spending in rural and urban areas of certain districts in Karnataka. A total of 61 households were interviewed over a period of two weeks in January 2024 using a semi-structured, validated interview guide with open-ended questions. Throughout the investigation, people’s perceptions were monitored.

### 2.1. Participant Recruitment and Profile

In this study, 61 participants aged 18 years and older were recruited through purposive sampling to explore the barriers to access reasons for out-of-pocket expenditure. The participants were recruited until data saturation, the point at which no new information was obtained. The participants were from rural areas of the Dakshina Kannada and Shimoga districts and both rural and urban areas of the Udupi district. The study included those individuals who recently incurred out-of-pocket expenditure, ensuring they have firsthand experience to share, and individuals who have accessed healthcare through different settings, such as hospitals, clinics, or private practitioners. Out of the 61 participants, 29 were male, and 32 were female (see Supplementary File S1: Participants’ profiles with hospital preferences). 

### 2.2. In-Depth Interviews

Sixty-one in-depth interviews were conducted with individuals who recently experienced out-of-pocket healthcare expenses and accessed healthcare through various settings. The interviews were conducted in Kannada and English for participants’ convenience at his or her residence. A verified in-depth interview guide was used to collect data through personal interviews. Interview guidelines were developed in English, but the interviews were held in Kannada, which was the native language of the participants. Before the main interviews, the interview guides were piloted with two participants to check for the flow and comprehensibility of the questions, probes, and timing of the interview questions. The interview guide began with opening questions followed by main questions and a few closing questions (see Supplementary File S2). The responses revealed that more OOPE was caused by medicines, diagnostic tests, and private healthcare services. The IDI guide focused on OOPE, the quality of healthcare services provided by both government and private hospitals, and insurance awareness. For instance, questions like ‘Why do you prefer to go to a private hospital or clinic for healthcare services’ were asked. The data were collected in rural areas of the Dakshina Kannada and Shimoga districts, and both rural and urban areas of the Udupi district during January 2024. Written and oral informed consent has been obtained from the participants for publication of the case report. The interviews were audio recorded, and the records were fully anonymized while the data were analyzed.

### 2.3. Data Analysis

The recorded data were transcribed and then translated into the English language for textual analysis. The names of the participants have been anonymized in order to protect their privacy. The interviews were analyzed with the help of Atlas.ti software version 7 to accomplish the coding process. A thematic analysis approach was adopted to underpin the study. In this analysis, codes were developed from the data based on the conceptual framework and objectives of the study and directly from the texts (participants’ views) themselves. These codes are the key themes to explain participants’ opinions on high out-of-pocket healthcare expenses. In the second stage, codes were merged and grouped to develop code groups or code families based on common characteristics for further analysis. We developed code families related to reasons for high healthcare OOPE, awareness of health insurance within communities in rural and urban areas of Karnataka, quality of services provided by government and private hospitals, and recommendations to reduce out-of-pocket healthcare expenses based on our research objectives (see Table 3 for code families and codes with example quotations). Each code was described by comparing different statements and quotes made by the participants.

### 2.4. Ethical Consideration

The Institutional Ethics Committee has approved this study, with reference numbers IEC 651/2023 for Dakshina Kannada, IEC 647/2023 for Shimoga, IEC 625/2023 for Udupi urban, and IEC 652/2023 for Udupi rural areas. Permission was received to conduct the study from the study area and institution. The purpose of the study and the method of data collection was explained to the participants and their identity was kept confidential.

## 3. Results

The results have been categorized into six different themes, viz., (1) Quality of care and infrastructure in government and private hospitals, (2) Affordability and accessibility of healthcare services, (3) Sources of healthcare expenses, (4) Awareness and utilization of health insurance, (5) Challenges in government healthcare services, and (6) Impact of health insurance on out-of-pocket expenses (OOPE).

### 3.1. Quality of Care and Infrastructure in Government and Private Hospitals

Participants had varying perspectives on their experiences at government hospitals. While some complimented favorable features such as competent service delivery, lower drug costs compared to private pharmacies, and access to free treatment, others were dissatisfied and encountered different difficulties.

“*Initially, government hospitals were not that good and clean. But now, it has improved because of the proper infrastructure and cleanliness*.”

(Jonathan, 25 years old, male, rural)

Some participants reported having a pleasant experience in private hospitals with high-quality services, and the majority of them stated that the therapy was both successful and resulted in immediate apparent changes in their health. In contrast, the disadvantages listed were mostly related to expenses and long wait times.

“*I recently visited the Orthopedic Department at a private Hospital, where Dr. Abraham provided good care. He conducted a thorough examination, including an X-ray, and accurately diagnosed my condition. He then prescribed the appropriate medication*.”

(Ashma, 45 years old, female, urban)

Most participants preferred private hospitals over government hospitals, claiming easier access to healthcare services and faster recovery times. Some argued that prioritizing health over hospital costs justifies a preference for private hospitals. They observed that in private hospitals, doctors provide their patients undivided attention throughout their therapy, assuring thorough care.

“*Doctors at private hospitals completely focus on their patients throughout the treatment process*.”

(Bakthapa, 59 years old, male, rural)

The study discovered a clear preference for private hospitals over government ones. Although greater wait times were seen in private hospitals, participants cited superior service quality and faster treatment availability as the primary reasons for choosing the facility. The majority of the participants chose faster healthcare procedures and shorter recovery times, demonstrating a strong preference for private facilities.

“*Private hospitals generally offer better treatment and facilities compared to government hospitals.*”

(Uma, 55 years old, female, urban)

### 3.2. Affordability and Accessibility of Healthcare Services

Several participants indicated affordability as the main reason for obtaining care at government hospitals.

“*In government hospitals, most services are free, while they charge for each service at private hospitals. Medications are also available at a lower expense in government hospitals, and doctors’ consultations are free*.”

(Rohan, 26 years old, male, rural)

While private hospitals are frequently regarded as giving greater quality care compared to government facilities, the higher expenses, longer waiting times, complex administrative processes, crowding, and difficulty with payment and insurance coverage are significant impediments that a few patients encounter. Many of the participants indicated affordability as the main reason for obtaining care at government hospitals.

“*Yes, in private hospitals, I feel that facilities are costly. If it were not for the economic strain, private hospitals are better. But middle-class families might feel a certain degree of financial burden*.”

(Ganapa, 38 years old, female, rural)

### 3.3. Sources of Healthcare Expenses

Participants covered their hospital stays using a variety of methods, including income savings, reimbursements, and several government and private health insurance programs (AB-PMJAY, ESIS, Sampoorna Suraksha, Medi Assist, HDFC, and ICIC health insurance).

“*When I was admitted to the hospital, the treatment expenses were covered through my son’s ESI. However, additional expenses for transportation and accommodation amounted to 50,000 rupees, which were managed using salary savings*.”

(Ashok, 48 years old, female, rural)

Participants displayed a variety of techniques for financing healthcare costs, including health insurance, incurring OOPE solely for uncovered treatments, reimbursement, and salary savings. Some emphasized the affordability of government hospitals, maybe due to a notion of free or subsidized care.

“*We will manage major health expenses with insurance, whereas minor health expenses will be covered by salary*.”

(Prathap, 26 years old, male, rural)

Some individuals used personal savings, including salary and healthcare savings accounts, to cover hospital bills.

“*In the case of health-related expenses, we have managed them separately through savings, so it has not caused any problems*.”

(Balaraj, 75 years old, male, rural)

### 3.4. Awareness and Utilization of Health Insurance

Participants displayed varying levels of awareness regarding health insurance schemes. While some expressed limited knowledge, attributing it to a perceived lack of government transparency and information dissemination, others demonstrated specific familiarity with initiatives like Ayushman Bharat and Jyothi Sanjeevini. Interestingly, some participants even contemplated the potential benefits of utilizing such schemes in the future.

“*Not one bit of information is correct. I honestly do not comprehend it. Everyone talks about Ayushman Bharat, and I have heard some of it. They describe Jyothi Sanjeevini as being advantageous to government personnel, but we have yet to use any of the facilities and are unaware how to do so. They claim to have it, but we are not sure what it comprises. Therefore, the information presented is not correct*.”

(Malathi, 40-year-old, female, urban)

Most individuals reported having some type of health insurance. Participants typically referred to government insurance as their primary coverage. However, some participants opted for private insurance.

“*I have a G-Shankar and Manipal Arogya card, which provides OPD benefits and other benefits up to 50,000 rupees. Additionally, I possess a company medical card, offering benefits up to 2 lakh rupees. All family members are covered under these cards*.”

(Navneeth, 36 years old, male, urban)

For most participants, health insurance clearly helped them manage their medical bills. This resulted in a lower OOPE, as indicated by participant reports of insurance coverage for treatment, testing, and drug expenditures. Furthermore, participants described the procedure of using these benefits as simple and without hassle.

“*One year ago, I underwent a heart operation, and the expenses were covered by the Ayushman Bharat card, which was incredibly helpful*.”

(Satish, 76 years old, male, rural)

### 3.5. Challenges in Government Healthcare Services

Longer wait times, a shortage of doctors, worries about the quality of care, accessibility issues, and infrastructure restrictions were among the challenges that participants reported when obtaining government healthcare facilities.

“*Waiting problem is there as per situation and government hospital is a bit far*.”

(Shoba, 45 years old, female, rural)

Participants had limited experience of government healthcare institutions, with the majority having never visited one and preferring private healthcare services. While noting the financial accessibility provided by government hospitals, particularly for those who cannot afford private care, they saw these facilities as resource-constrained when compared to their private counterparts. This shows a possible trade-off between affordability and perceived quality of care, emphasizing the need for improved public understanding and communication about government healthcare programs.

“*We do not typically visit government hospitals, so I do not know much about them. However, since government hospitals often have poorer healthcare facilities, we tend to avoid them. Nevertheless, I acknowledge that government hospitals are beneficial as they provide free treatment*.”

(Somu, 70 years old, male, urban)

In contrast, some of the participants indicated varying opinions on the value of government healthcare institutions, especially Primary Health Centers (PHCs). Affordability surfaced as an important subject, with several participants appreciating decreasing expenses and the availability of free medication. Others expressed pleasure with the care received, emphasizing its efficacy.

“*Government hospitals are good. I visited during the Corona time, and all the facilities were excellent. They provided me with effective tablets, and I recovered well*.”

(Bhavya, 59 years old, female, urban)

### 3.6. Impact of Health Insurance on Out-of-Pocket Expenses (OOPE)

Participants’ perceptions of the elements that contribute to significant OOPE during hospitalizations varied. While some individuals were concerned about the total cost of hospital procedures, others cited specific cost drivers such as investigations or medications. Limitations in government insurance coverage surfaced as a key concern. One participant directly related greater OOPE to uncovered outpatient department charges, emphasizing the financial burden that comes with such constraints.

“*Everything seems to be getting more expensive, especially when it comes to hospital materials. For example, a single gauze pack costs around 60 Rs, and a single suture can cost between 600 to 700 Rs. Nearly every type of treatment is becoming increasingly expensive*.”

(Vinoda, 58 years old, female, urban)

Health insurance had an important part in lowering OOPE for healthcare and giving financial security to people and families. Participants reported considerable benefits from health insurance coverage, such as lower healthcare expenses, higher affordability, and easier access to medical care when seeking services.

“*Previously, we had a hospital bill of around 50,000, but as we had the card, we had to pay just 5000, so the health insurance has been helpful*.”

(Jaya, 38 years old, female, rural)

Most participants strongly supported health insurance to reduce the financial burden of healthcare bills. They voiced concerns about the escalating cost of medical services, recognizing the limitations of depending primarily on wage savings to cover unexpected healthcare costs. The unexpected nature of medical costs was another aspect, emphasizing the importance of having insurance to counter potential financial pressure and minimize OOPE.

“*Yes, I believe that everyone should have health insurance nowadays. You never know when you might need to visit the hospital or encounter a health issue, and medical expenses can be significant. While government hospitals may not always provide the necessary facilities, private hospitals can be quite costly. Therefore, having health insurance can be incredibly beneficial in such situations*.”

(36 years old, male, urban)

Participants unanimously supported health insurance as an approach to reduce OOPE. Several participants identified specific cost drivers in the healthcare system, citing diagnostic tests and drugs as major areas where cutting costs would dramatically reduce OOPE. These findings imply that participants had a common awareness of the necessity of health insurance and the need for targeted measures to address key cost drivers in the healthcare system.

“*If the government reduces taxes slightly, the cost of important medicines may decrease. This would make these medications more affordable for people, ultimately reducing the overall expense of healthcare*.”

(Ajith, 40 years old, female, urban)

## 4. Discussion

This study examined the critical determinants of OOPE in Karnataka, India, focusing on why people prefer private hospitals over public healthcare services. It identified significant disparities: lower-income households often use government hospitals for their affordability and free medications, while higher-income individuals prefer private hospitals for better facilities and doctor choice. This highlights the financial strain and access challenges faced by different socioeconomic groups.

These findings align with previous research, which identified income level as a significant factor influencing healthcare-seeking behavior and OOPE. Their study reported that financial constraints lead lower-income groups to utilize government services more frequently, while higher-income groups prioritize perceived superior quality by opting for private healthcare [15]. The study revealed that there are differing perceptions of treatment quality and infrastructure across government and private hospital settings [11]. Participants noted improvements in government hospitals, noting increased cleanliness and better amenities, which is consistent with recent government initiatives. However, the demand for private hospitals, fueled by beliefs of superior quality care, personalized attention, and faster recovery times, frequently outweighed these advancements. Private hospitals typically offer better amenities and faster service, despite higher expenses [47]. This emphasizes the need for government hospitals to improve patient-centered treatment and minimize wait times to compete effectively. Affordability appeared as a major factor influencing healthcare decisions, with participants favoring government hospitals due to cheaper expenses and free treatments, particularly among low-income individuals. This is consistent with observations that the cost of care leads people to public hospitals, despite quality difficulties. Private hospitals, on the other hand, were preferred despite greater costs for perceived improved quality of care and shorter wait times, which justified the additional expense to many participants. This duality reflects broader trends, highlighting the need for governmental actions to enhance the affordability and quality of public healthcare [48,49]. Participants used a combination of income savings, reimbursements, and health insurance to meet healthcare bills, demonstrating a variety of medical cost-management techniques. The considerable reliance on savings emphasizes the financial strain on households without adequate insurance coverage, as out-of-pocket expenses, to be a major barrier to healthcare access [50,51]. The importance of health insurance and personal savings in reducing costs has also been emphasized. This recommends that more people should be aware of and have access to comprehensive health insurance plans to alleviate the financial strain on individuals and families. There was a noticeable difference in participants’ awareness and use of health insurance schemes. While some benefited from government and private plans, others lacked knowledge, particularly of government initiatives such as Ayushman Bharat. However, it is important to acknowledge that participant perspectives on health insurance are not always uniform. In another study, some participants expressed less satisfaction with their health insurance, while others mentioned it as a source of relief for dealing with hospitalized family members [52]. In a different study, some participants felt that they should receive their full reimbursement at the end of the year if they stayed healthy for the entire year [53]. Participants identified issues with government healthcare services, such as higher wait times, limited accessibility, and a perceived worse quality of care. These difficulties, combined with infrastructure limits, frequently resulted in a preference for private hospitals, despite the greater expenses. The Janani Suraksha Yojana (JSY) has positively impacted reducing financial burdens, but further program enhancements are needed to address complex obstetric care needs in Rajasthan [54]. A separate study highlights similar challenges faced by other public health initiatives, including inadequate financial incentives for participation, delayed payments, difficulties with residency verification, and excessive administrative paperwork. These factors can contribute to lower program acceptance rate [55].

Health insurance was highlighted as critical for minimizing OOPE and providing financial security. Participants cited considerable cost-saving benefits that reduced the financial burden of hospital stays and treatments [56]. This highlights the need for more comprehensive insurance that covers a broader variety of expenses, as well as specific initiatives to cut the cost of diagnostic tests and medications to make healthcare more accessible.

## 5. Conclusions

The study highlights the different perspectives between government and private hospitals while highlighting the complex terrain of healthcare choices and experiences among participants. On the one hand, people from lower socioeconomic backgrounds value government hospitals because they are accessible and affordable. Participants saw that government hospitals now had better facilities and cleaner facilities, which improved care delivery and reduced prescription prices. Significant obstacles still exist, though, including prolonged wait periods, a lack of medical personnel, and worries about the standard of care. Despite their greater costs, these disadvantages frequently encourage people to use private hospitals. On the other hand, patients choose private hospitals because of their better quality of care, quicker recovery periods, and committed medical staff. However, the drawbacks of private hospitals include expensive costs and complicated administrative procedures. The preference for private hospitals highlights a stark difference in how people view the quality of care, with many respondents prepared to pay more for quicker and better care.

The findings also highlight how important health insurance is for reducing out-of-pocket costs (OOPE) and improving access to treatment. There were differences in the participants’ knowledge and use of health insurance plans, and many of them were taking advantage of both public and private insurance. It was generally acknowledged that health insurance was an essential instrument for controlling medical costs, alleviating financial stress, and granting access to essential healthcare services. In spite of this, many continue to experience considerable OOPE due to gaps in insurance coverage, particularly for outpatient therapies. According to the study, there is an overall support for more extensive health insurance coverage in order to lessen the financial strain on individuals and families. In addition, the participants conveyed their wish for more government outreach and information sharing on existing health insurance plans, highlighting the necessity of raising public awareness and educating the public. This thorough analysis of healthcare preferences, affordability issues, and the function of health insurance emphasizes the necessity of focused policy changes to raise the standard of care, increase accessibility, and safeguard everyone’s financial interests.

## Figures and Tables

**Table 1:** Number of (CHCs), (PHCs), and (SCs) in Karnataka Rural [19].

Rural	Required (R)	In Place (P)	Shortfall/Excess (S) (%)
Number of Community Health Centers (CHCs)	329	189	42.55 Shortfall
Number of Primary Health Centers (PHCs)	1318	2176	65.1 Excess
Number of Sub-Centers (SCs)	8024	9188	14.51 Excess

**Table 2:** The OOPE for IPD care per hospitalized case [19].

Facility	Rural Karnataka	Urban Karnataka
IPD—Per hospitalized case (in Rs.)—Public	4719	5451
IPD—Per hospitalized case (in Rs.)—Private	18,120	27,560

**Table 3:** Code families and codes with example quotations.

Code Family	Code	Example Quotations
Awareness	Lack of knowledge	If you go to a government hospital, you can give priority to it because there is always benefit to the people, but I have not been to a government hospital so far, so I have no knowledge. There is no benefit. So far, I have been using private insurance since I have Medicare. (Participant 2, Location: Udupi)
Coping strategies for health expenditure	Financial management	We have all the cards for hospital visit, if we go private, we use that, if we go government, it will be free. (Participant 1, Location: Udupi)
Insurance	Benefits of Health Insurance	If insurance is there, if anyone gets admitted to the hospital, it will cover expenses, and it will claim immediately. There is no problem with waiting, so I recommend insurance to everyone. (Participant 11, Location: Dakshina Kannada)
Out-of-pocket-expenditure	Preplanning to reduce OOPE	We should give first preference to health and spread awareness about Ayushman Bharat because it can reduce hospital expenses. For some people who cannot afford big hospitals, insurance will be helpful in such cases. (Participant: 2, Location: Dakshina Kannada)

## Data Availability

Data is available from the corresponding authors on reasonable request.

## Supplementary Materials

The following supporting information can be downloaded at https://www.mdpi.com/article/10.3390/ijerph21101287/s1, File S1: Participants profile with hospital preferences; File S2: Interview Guide.
