# Supplementary material for: “Private Hospitals Generally Offer Better Treatment and Facilities”: Out-of-Pocket Expenditure on Healthcare and the Preference for Private Healthcare Providers in South India
Source: Int J Environ Res Public Health. 2024 Sep 26;21(10):1287. doi: 10.3390/ijerph21101287 (PMC11508021; doi:10.3390/ijerph21101287)
Supplement: Supplementary file 1 [file ijerph-21-01287-s001.zip › ijerph-3144455-supplementary-1.pdf]

Supplementary File S1: Participants profile with hospital preferences

Participants	Names *	Gender	Age	Place	Hospital Preference	Insurance
**P1**	Suji	F	48	Parkala	Private	ESI & Manipal health card
**P2**	Sanju	M	73	Parkala	Private	Manipal Medicare
**P3**	Uma	F	55	Shettibettu	Private	ESI & Manipal Medicare
**P4**	Somu	M	70	Parkala	Private	Star health & G Shankar card
**P5**	Malathi	F	40	Shettibettu	Private	Star health
**P6**	Vinoda	F	58	Golikatte,	Private	Manipal Medicare
**P7**	Arathi	F	51	Herga	Private	Manipal Medicare
**P8**	Manju	M	58	Herga	Private	Manipal Medicare card and SBI Insurance
**P9**	Ramesh	M	52	Golikatte	Private	ESI & Gshankar card
**P10**	Ajith	M	36	Herga	Private	Gshankar & Private health insurance
**P11**	Navneeth	M	36	Parkala	Private	Star health & Gshankar card
**P12**	Ashma	F	45	Shettibettu	Private	Manipal health card & Manipal Medicare

**P13**	Sathish	M	50	Kodange	Government	Manipal health card & Nagarasabhe card
**P14**	Bhavya	F	59	Parkala	Government	Konkani card
**P15**	Sugandhi	F	36	Herga	Private	ESI, Ayushman card & Manipal health card
**P16**	Satish	M	76	Sogane	Both private & government	Ayushman Bharat
**P17**	Balaraj	M	75	Shimoga	Both private & government	Ayushman Bharat
**P18**	Somesh	M	63	Sogane	Private	Medi assist
**P19**	Veena	F	50	Shimoga	Private	Ayushman Bharat, ESI
**P20**	Shamla	F	49	Sogane	Government	Ayushman Bharat
**P21**	Mala	F	48	Shettyhalli	Private	ESI, LIC
**P22**	Bakthapa	M	59	Ayanuru	Private	Reimbursement
**P23**	Ananth	M	61	Shettyhalli	Private	Reimbursement
**P24**	Rachana	F	30	Ayanuru	Private	LIC, Reliance health insurance
**P25**	Murthy	M	62	Sogane	Private	Medi assist
**P26**	Sujatha	F	40	Shettyhalli	Private	LIC (for children only)
**P27**	Shoba	F	45	Shettyhalli	Private	LIC
**P28**	Reshma	F	48	Shettyhalli	Government	ESI
**P29**	Virbhadra	M	66	Shettyhalli	Private	Star health insurance

**P30**	Lacchu	F	43	Ayanuru	Government	CGHS
**P31**	Pushpa	F	39	Sogane	Government	Ayushman Bharat, Sampoorna Suraksha
**P32**	Yashu	F	50	Chelairu	Private	Sampoorna Suraksha, ESI
**P33**	Vinitha	F	54	Chelairu	Government	Mediclaim, Ayushman card
**P34**	Jaya	M	32	Kalladka	Private	Ayushman, Sampoorna Suraksha
**P35**	Ashwath	M	24	Hosabettu	Government	Star Health
**P36**	Chandra	F	51	Hosabettu	Both private & government	Yeshashwini
**P37**	Shruthi	F	38	Hosabettu	Private	Yeshashwini
**P38**	Pradeep	M	58	Hosabettu	Both private & government	Private Insurance
**P39**	Ashok	M	48	Hosabettu	Private	Future general insurance
**P40**	Ravi	M	23	Hosabettu	Government	Mediclaim
**P41**	Ganapa	M	38	Chelairu	Private	Star Health, ICICI Lombard insurance
**P42**	Savitha	F	45	Chelairu	Government	Ayushman, Sampoorna Suraksha

**P43**	Prathap	M	26	Vamanjoor	Private	Liberty insurance
**P44**	Rohan	M	26	Vamanjoor	Private	HDFC insurance
**P45**	Rasmi	F	32	Kuntala Gudda	Private	Medicare
**P46**	Pranith	M	25	Vamanjoor	Private	ICICI Lombard
**P47**	Jaya	F	38	80th Badagabettu,	Government	Ayushman Bharat and ESI
**P48**	Jovitha	F	54	Nadidhare	Government	Ayushman Bharat
**P49**	Janvi	F	45	80th Badagabettu	Private	Ayushman Bharat
**P50**	Juniper	F	32	Nadidhare	Private	Ayushman Bharat and ESI
**P51**	Jayanthi	F	34	80th Badagabettu	Private	Ayushman Bharat
**P52**	Jairam	M	36	Sanakibettu	Government	ESI
**P53**	Joseph	M	47	80th Badagabettu,	Government	Ayushman Bharat
**P54**	Junaeed	M	30	Alambi	Private	Ayushman Bharat and ESI
**P55**	Jayshree	F	23	Sanakibettu	Government	ESI, Arogya Karnataka, Yeshashwini

**P56**	Jonathan	M	25	Sanakibettu	Private	ESI, Ayushman Bharat, Ye-shashwini
**P57**	Javier	M	45	Bottikambala House	Government	ESI, Ayushman Bharat, Ye-shashwini
**P58**	Jeswanti	F	48	80th Badagabettu	Government	Ayushman Bharat and ESI
**P59**	Judith	F	46	Vijaya gar-den	Private	ESI, Ayushman Bharat, Ye-shashwini
**P60**	Jameela	F	40	H.N. Mrinal. 3/207(B), 80th Badagabettu.	Private	Ayushman Bharat and ESI
**P61**	Jagan	M	28	Nallur house, 80th Badagabettu.	Private	Ayushman Bharat and ESI

(* All original names are anonymized)
