# Supplementary material for: “Private Hospitals Generally Offer Better Treatment and Facilities”: Out-of-Pocket Expenditure on Healthcare and the Preference for Private Healthcare Providers in South India
Source: Int J Environ Res Public Health. 2024 Sep 26;21(10):1287. doi: 10.3390/ijerph21101287 (PMC11508021; doi:10.3390/ijerph21101287)
Supplement: Supplementary file 1 [file ijerph-21-01287-s001.zip › ijerph-3144455-supplementary-2.pdf]

INTERVIEW GUIDE

SOCIO-DEMOGRAPHIC DETAILS:

1. No. of family members: _____
   No. of Male family members: _____
   No. of Female family members: _____

Age	
Gender	
Marital status	
Education	
Occupation	

2. Number of dependents in the family:
3. Type of ration card:
4. Annual income:
5. Monthly medical expenditure:
6. Nearest government healthcare facility:
7. Distance to the nearest government healthcare facility.
8. Nearest private healthcare facility
9. Distance to the nearest private healthcare facility.
10. What is the health facility you prefer:
11. Have you heard about any government insurance schemes? Can you name some?

1. In the last 5 years, did you or anyone in your family need medical care?
PB1: If yes, what was the purpose? How were the financial aspects managed?
PB2: If no, have you ever thought of how you would handle such a situation?

2. What is your opinion about healthcare services by the government (centre/state)?
PB1: If you need access to a healthcare facility, will you choose a government hospital/government pharmacy? Why/why not?
PB2: Can you share your experience regarding a recent visit to the hospital?

3. Did you face any challenges in accessing healthcare services?
Hint: (e.g. distance, transportation, stay, waiting time, cost, language barriers)
PB1: Did you encounter any challenges during the visit?
PB2: What were some benefits of government hospitals you have observed?
(hint: quality of services, infrastructure)

4. How do you manage health-related expenses at home?
PB1: What are the sources of financial support you seek to manage health?
PB2: How have health-related aspects impacted your household finances?

5. Have you ever felt that medical expenses are going out of your control?
PB1: Were you ever burdened by your payments for medicines and healthcare?

6. What do you think are the reasons for higher expenditure on health?

7a. What do you believe are effective ways to reduce hospital expenses?
7b. Do you have any health insurance?
i. If yes, which health insurance?
ii. What is the annual premium that you pay?
iii. What is the sum insured?
iv. How many members of the family are covered?

8. What impact has health insurance had on your health expenditure?
PB1: Are there any specific challenges or difficulties that you have faced when it comes to utilizing the benefits of health insurance?
PB2: If not insured, what do you think is stopping you from being insured?

9. Do you recommend health insurance as a way to overcome the burden of healthcare at home? [Y/N]
PB1: Why do you think so?
